# Epigenetic Regulation of Telomere Maintenance for Therapeutic Interventions in Gliomas

**DOI:** 10.3390/genes8050145

**Published:** 2017-05-17

**Authors:** Elisabeth Naderlinger, Klaus Holzmann

**Affiliations:** Institute of Cancer Research, Comprehensive Cancer Center, Medical University of Vienna, Borschkegasse 8a, Vienna 1090, Austria; elisabeth.naderlinger@gmx.at

**Keywords:** telomere maintenance, high grade glioma, epigenetic therapy, CpG DNA methylation, chromatin modification, histone methylation and acetylation, histone deacetylase, miRNA, telomeric repeat-containing RNA

## Abstract

High-grade astrocytoma of WHO grade 4 termed glioblastoma multiforme (GBM) is a common human brain tumor with poor patient outcome. Astrocytoma demonstrates two known telomere maintenance mechanisms (TMMs) based on telomerase activity (TA) and on alternative lengthening of telomeres (ALT). ALT is associated with lower tumor grades and better outcome. In contrast to ALT, regulation of TA in tumors by direct mutation and epigenetic activation of the hTERT promoter is well established. Here, we summarize the genetic background of TMMs in non-malignant cells and in cancer, in addition to clinical and pathological features of gliomas. Furthermore, we present new evidence for epigenetic mechanisms (EMs) involved in regulation of ALT and TA with special emphasis on human diffuse gliomas as potential therapeutic drug targets. We discuss the role of TMM associated telomeric chromatin factors such as DNA and histone modifying enzymes and non-coding RNAs including microRNAs and long telomeric TERRA transcripts.

## 1. Introduction

Genetic information and the pattern in which genes are expressed are both important for properties of cells. Human cell types show broad diversity and specialization although, in a given person, cells share identical genetic information. Gene expression patterns important for cell phenotype and function need to be both adaptable and heritable. Adaptability allows that specialized cell types and functions such as telomere maintenance mechanisms (TMMs) arise from common cell precursors in response to specific signals from the environment, whereas heritability ensures that the integrity of cell type lineages can be maintained through cell divisions. Adaptable gene expression patterns are often responses to stimulation and based on non-genetic determinants that are summarized as epigenetic mechanisms (EMs) with fundamental implications for cancer especially in combination with heritable mutations [1,2]. These EMs currently include covalent modification of DNA, covalent modification of histones, non-protein-coding RNAs (ncRNAs) such as short microRNAs (miRNAs) and long non-coding RNAs (lncRNAs). Genetic mutations that target epigenetic modifiers of EMs probably cause genome-wide epigenetic alteration in cancer (reviewed in [2]).

EMs are important for providing the proper regulation of telomerase activity (TA) in several biological states, such as embryonic down-regulation of the limiting factor hTERT contributing to aging and upregulation of hTERT to gain immortality in most cancers [3]. Moreover, EMs form condensed heterochromatin structures at telomeres and subtelomeres densely compacted by repressive DNA methylation and histone modifications [4]. Differential abundance of epigenetic modifications at telomeres and subtelomeres contributes to the formation of chromatin state termed as closed or open, which regulates telomere length possibly through regulating the access of telomerase to telomeres or the less common alternative lengthening of telomeres (ALT) mechanism.

In this review, we provide an overview of epigenetic regulation and possible therapeutic interventions of ALT identified in normal and cancer cells with a focus on diffuse gliomas as one special group of tumors located in the brain with diverse patient outcome depending on TMM used.

## 2. Telomere Maintenance Mechanisms

### 2.1. TMM in Pluripotent Non-Malignant Cells

Telomeres are conserved nucleoprotein structures in eukaryotic cells and are localized at the ends of all linear chromosomes preventing DNA damage response (DDR) and progressive loss of genomic information caused by semi-conservative replication of DNA [5]. The shelterin protein complex consisting of six telomere-specific proteins recognizes and assembles the telomeric DNA configuration to shape and safeguard telomeres [6,7]. Human somatic cells do not demonstrate a TMM and reach with shortened telomeres a limit of proliferation capacity termed replicative senescence [8,9]. Beside well-established telomere shortening as the replication counting mechanism, accumulation of epigenetic changes may act as a mitotic clock by progressive loss of total DNA methylation and decrease of heterochromatin domain stability [10,11]. Evidence suggests that the primary function of senescence may be an antiviral defense mechanism as many viruses have developed proteins that prevent senescence of the cells they infect [12]. Senescence together with apoptosis suppresses the development of cancer but may also induce a decline in the replicative function of certain stem-cell types that contribute to mammalian aging [13]. Telomere length in stem cells is established long enough during embryogenesis to ensure tissue homeostasis but short enough to limit cell proliferation capacity and cancer initiation [14]. Such optimal telomere length is observed in human embryonic stem cells that demonstrate stable telomere length important for genomic stability [15]. The mechanism controlling telomere length in human embryonic and induced pluripotent stem cells involves active telomere trimming in addition to elongation by telomerase activity [16]. ALT is a further TMM without TA based on homologous recombination (HR) and homology-directed telomere synthesis that is identified in non-diseased mouse tissues [17,18] and some mouse stem cells [19]. Evidence from recent studies suggests that ALT may exist in human endothelial, stromal and some epithelial cells of a wide variety of non-neoplastic tissues, including placenta [20,21]. In summary, the available data from human and rodents demonstrate that either TA or ALT exist in specialized pluripotent cell types but are absent in somatic cells. The presence of TMMs in these cells supports the requirement for telomere length maintenance in the germ line also to prevent intergenerational telomere loss. Furthermore, it is speculated that ALT may be a persistent component of telomere biology that coexists already with TA as a back-up TMM during evolution in most eukaryotes [22].

### 2.2. TMM in Cancer

Reactivation of a TMM in incipient cancer cells is an important key factor for tumors to survive cellular crisis and gain endless proliferation capacity [23]. Immortality of cells is thus one of the essential hallmarks of cancer [24,25]. In general, TA is observed in over 80% and ALT in 10–15% of human cancer types [26].

Since the discovery of the enzyme telomerase in ciliates [27], this activity was investigated in great detail at the molecular and cell biology level in many eukaryotic species [28,29,30]. The enzyme telomerase adds new telomere sequences to the distal ends of chromosomes via addition of units of the repeated telomere core sequence each consisting of six nucleotides with the sequence TTAGGG [31].

The main component of telomerase in humans is a ribonucleoprotein that consists of the catalytic protein telomerase-reverse-transcriptase (hTERT) and the telomerase-RNA-component (termed hTR or hTERC), and additional cofactors [23,32]. While hTR is generally expressed in cells, the hTERT gene expression and TA become reactivated in cancer cells by mechanisms that include chromosomal rearrangement, promoter mutation [33] and epigenetics [3,34]. However, there is evidence that levels of hTERT and hTR are both limiting for TA in tumors including gliomas (reviewed in [35,36]).

One or more alternative mechanisms for the lengthening of telomeres other than telomerase were identified in immortalized fibroblasts, tumor cell lines and tumors [37,38,39], reviewed in [40]. About 25% to 60% of sarcomas and only about 5% to 15% of carcinomas show ALT [41]. ALT has shown high prevalence in sarcomas but also in astrocytoma [26]. The higher prevalence in tumors of mesenchymal or neuroepithelial origin could be due to cell-type specific mechanisms promoting ALT instead of telomerase that include epigenetic modifications at telomeres [42,43]. ALT can be deduced from presence of TMM in the absence of TA. Phenotypic characteristics of ALT activity in contrast to TA include very long but heterogeneous telomere length as first recognized in tumor cell lines [39]. Phenotypic markers commonly used for ALT activity are ALT-associated PML bodies (APBs), telomeric and GC-rich minisatellite instability, telomeric-sister chromatid exchange (T-SCE), and extrachromosomal telomeric DNA [41]. Extrachromosomal telomeric repeat (ECTR) DNA is very abundant in ALT cells and can be linear or circular. Double stranded telomeric DNA circles and partially single stranded C- or G-rich circles occur with the C-Circles being most specific for detection of ALT cells. Indeed, the C-Circle Assay (CCA) represents a robust and quantitative ALT assay that responds quickly to changes in ALT activity e.g., induced by gamma-irradiation [44]. ALT occurs more often in tumors with complex karyotype due to telomere dysfunction-driven chromosomal instability in neoplasia (CIN) and is dependent on HR that requires the integrity of the MRN (MRE11-RAD50-NBS1) recombination complex [45,46]. The deciphering of further molecular details appears challenging. Several candidate genes/proteins that are required for ALT were identified as components of telomere chromatin, APB and DNA metabolisms including HR (reviewed in [47]). Examples include heterochromatin protein HP1 isoforms as components of APBs and shelterin proteins TRF1, TRF2, POT1, TIN2 and RAP1. Regulation by phosphorylation of TRF1 on specific site threonine 271 was recently identified as necessary for APB formation in ALT cells [48]. In addition, recent studies demonstrated that recurrent mutations of the Alpha Thalassemia/Mental Retardation Syndrome X-Linked (ATRX) or Death-Domain Associated Protein (DAXX) genes encoding histone-chaperons influence ALT activation and maintenance (reviewed in [49]). ATRX in a complex with DAXX and Histone H3.3 encoded in H3F3A gene is important for heterochromatin silencing to maintain the H3K9me3 modification at multiple genomic regions such as tandem repeats at telomeres and centromeres and interspersed repeats, e.g., LINEs, SINEs, Alu repeats and endogenous retroviral repeats (ERVs) (reviewed in [50]). Mutations in all three genes, ATRX, DAXX and H3F3A, of this pathway are very often observed associated with mutations in TP53 in cancer cells using ALT as TMM [51,52]. RNAi-knockdown of ATRX in human immortalized cell cultures, telomerase positive tumor cells and mouse embryonic stem cells by itself is not sufficient to produce ALT, suggesting that additional genetic/epigenetic changes are required to initiate ALT [53,54,55]. Indeed, results from knockout of ATRX in human mortal cells or immortal telomerase-positive cells showed that deactivated ATRX is insufficient to activate ALT [56]. ATRX loss in SV40-transformed mortal fibroblasts increased the proportion of cell lines activating ALT instead of TA and decreased the time prior ALT activation. Moreover, transient ATRX expression repressed ALT activity in cells using ALT as TMM. These findings are in line with the prior results that loss of ATRX function is necessary but not sufficient for activation of ALT. Another study showed that loss of the histone chaperone ASF1 led to the acquisition of several ALT phenotypes and thus suggesting that ALT is established via a multistep process [57]. Indeed, in vitro switch of TA to ALT activity in telomerase-positive cancer cells was successfully demonstrated by inducing telomere-specific DNA damage, ATRX and DAXX knockdown, and deletion of hTERT [58]. ALT activity measured by CCA from a mouse model of ATRX-deficient GBM was found in DNA extracted from a subset (3 of 8) of tumors and neurospheres but not normal brain samples [59]. These in vivo mouse data further demonstrated that ATRX loss promotes tumor growth and impairs non-homologous end joining DNA repair in glioma resulting in genetically unstable tumors that may support activation of ALT.

It was very long hypothesized that ALT functions via homology-directed telomere synthesis (reviewed in [22]). Recent studies support this suggestion of ALT as a conservative DNA replication process of human telomeres with features of break-induced replication [60] and that break-induced telomere synthesis underlies ALT [61]. Another study reported that ALT preferentially occurs at telomeric lagging strands and thus explaining heterogeneous telomere lengths observed in most ALT cancers [62].

To summarize, two types of templates are available for synthesizing de novo telomere DNA by TMMs to compensate for the telomeric attrition due to cellular replication. While ALT requires copying of DNA templates, TA uses a short RNA template and reverse transcription. Recent data from TMM analyses without evidence for TA or ALT activity indicate that additional mechanism(s) in tumors with clinical relevance may exist and are termed as non-defined telomere maintenance mechanism (NDTMM) [63,64,65]. Telomeres are transcribed to telomeric repeat-containing RNA (TERRA) as long ncRNA transcripts [66,67] and it is therefore tempting to speculate that novel telomeric synthesis mechanisms may use such templates. In line with this point is the recent finding of telomeric repeat containing dsRNA which can act as template for de novo telomere addition in ciliates during macronuclear differentiation [68].

### 2.3. TMM and Clinical Outcome

TA or subunit transcript expressions are generally associated with poor prognosis in breast and colorectal cancer, but for patients with ALT, the prognosis varies among different tumor types of sarcoma and astrocytoma, where ALT shows high prevalence (reviewed in [36,40]). In osteosarcoma, malignant fibrous histiocytomas and liposarcoma ALT was associated with decreased survival than was TA. These adverse findings might not be specific for human cancers as we recently identified ALT occurring with high prevalence in more aggressive types of canine sarcomas [64]. In contrast, presence of ALT in glioblastoma multiforme (GBM) was associated with better patient outcome [69,70,71], whereas in pediatric high grade gliomas TA confers poor outcome [72]. ALT shows higher prevalence in astrocytoma of lower grades 2 and 3 compared to grade 4 GBM. It is suggested that the differential prognostic significance of ALT in gliomas may depend on the different genetic and epigenetic events responsible for activation of TMMs that is specific for the cell of origin. Furthermore, both TMMs may drive tumor progression, e.g., telomerase has non-canonical functions that support other hallmarks of cancer [73,74], and ALT has been associated with complex karyotype and chromosomal instability (reviewed in [36]). Prognosis as well as the genetic background of different ALT tumor types varies substantially, e.g., ALT gliomas and sarcomas frequently lose ATRX (reviewed in [49]), whereas pancreatic neuroendocrine tumors that use ALT have frequent DAXX mutations, but other ALT tumors do not associate with loss of ATRX/DAXX [75,76,77].

A subset of tumors with neither TA nor evidence of ALT may exist in a number of tumor types although immortalization is considered as an essential hallmark of cancer (reviewed in [36]). NDTMM tumors were more frequently identified in subgroups of GBM patients with CDKN2A G500 allele and are associated with poor outcome [65]. GBM subgroups with NDTMM showed an increased immune signature compared with tumors using ALT or TA and it was suggested that specific tumor-associated macrophages are more unfavorable [63]. High prevalence of tumors with NDTMM in GBM subgroups is supported by our published finding in a cohort of primary GBMs of 50% cases with TA [78], and has been recently further characterized for ALT specific C-Circles resulting in only 7% with ALT [79]. Our results agree with similar prevalences of 15–17% ALT cases in GBM (reviewed in [40]) and raise now important concerns over therapeutics targeting TMMs, which are not expected to be effective against TMM negative tumors. It is currently not clear if this is due to false negative TMM assay results or to the presence of another ALT mechanism, or whether these tumors do not use any TMM (reviewed in [36]).

### 2.4. TMM Cell Models for Glioma

Clinical associations as described before between TMM and outcome of glioma patients are supported by following results from preclinical studies. ALT in tumor derived cell lines and immortalized fibroblasts is associated with extreme long and heterogeneous telomeres, defects in the G2/M checkpoint of the cell cycle and reduced capability of double-strand break repair [54]. These attributes may facilitate treatment of ALT positive cancers and thus better explain outcome in gliomas. However, the number of established in vitro glioma cell models with ALT is currently very limited and thus hamper detailed comparative in vitro studies, e.g., important for drug screenings targeting TMMs in gliomas.

As described before switching of TA to ALT activity in a fibrosarcoma cell line model derived from HT1080 was recently demonstrated in vitro [58], but it remains to be validated if this approach is generally applicable for TA cells including those from glioma.

Tumor stem cells involved in therapy resistance use ALT for sustained long-term proliferation as demonstrated with a TG20 termed promising ALT cell line model of glioma stem cells (GSCs) established from GBM neurosphere cultures [80]. Established TG20 cells were more resistant to ionizing radiation than GSCs with TA and it was suggested that this resistance was related to interference of ALT pathway with DDR. TG20 cell line shows all features of cancer stem cells, such as the expression of neural stem cell markers, the generation of intracerebral tumors in immune-deficient mice as well as in nude mice, and the ability to sustain serial intracerebral transplantations without expressing telomerase. Intracerebral grafts of TG20 cells in mice were shown to represent a valuable preclinical GBM model of ALT for potential therapy screening [81].

A specific cell line model JHH-273 was reported, that does not proliferate in vitro but grows in both the flank and orthotopically in the mouse [82]. This model shows genome hypermethylation as epigenetic features and characteristic of IDH1 mutated anaplastic astrocytoma with concurrent mutations in TP53, CDKN2A and ATRX.

We recently identified glioma cell lines with long telomeres and absence or low TA [78]. Candidate ALT cell lines were screened for ALT associated C-Circles and one TA negative cell line YTBO was identified as ALT GBM cell model [83]. This cell line was established from a patient derived glioma tissue of a distinct morphological subset of GBMs and was cultivated in medium containing serum. YTBO cells demonstrate beside telomere C-Circles further essential features characteristic of ALT cells, such as long and heterogeneous telomeres, APBs containing telomeric DNA and shows reduced in vivo tumorigenicity in immune-deficient mice compared to other TA positive GBM cell lines.

## 3. Diffuse Gliomas

### 3.1. Incidence

Brain tumors are a heterogeneous group of benign or malignant neoplasms within the central nervous system [84,85]. For example, in Austria, the overall incidence rate is around 18 cases per 100,000 people per year according to the established Austrian Brain Tumor Registry [86]. Of approximately 1700 primary brain tumors newly diagnosed in Austria per year, 51% are classified as non-malignant and 49% as malignant tumors. The vast majority of intracranial tumor entities are sporadic. Brain tumors are classified according to histopathological criteria into four different grades by the terminology and definitions of the World Health Organization (WHO) [84] and since 2016 the update classification uses molecular parameters in addition to histology to define many tumor entities [87].

As gliomas account for approximately 30% of all brain neoplasms and 80% of all malignant brain tumors, these neoplasms are of special relevance in routine clinical practice [88]. Three main histopathological subtypes including astrocytomas, oligodendrogliomas and mixed oligoastrocytomas are distinguished according to the WHO criteria. Tumors with an oligodendroglial component are generally associated with a better patient prognosis. The origins of malignant gliomas are the result of different pathways originating in multiple sources that all ultimately converge in the same disease [89]. Recent studies support the notion that neural stem cell (NSC), astrocyte and oligodendrocyte precursor cell (OPC) can all serve as the cell of origin with implication in the development of effective therapeutic treatment methods [90].

### 3.2. Diagnostics

Diffusely infiltrating gliomas with WHO grades II to IV represent the most common type of glioma and are characterized by their diffusely infiltrative growth into the normal brain tissue [84,87]. WHO grade IV gliomas are termed as GBM and represent both the most common primary brain tumors and the most aggressive glioma type as well [91,92]. Generally, GBMs consist of a necrotic tumor center that is surrounded by compact malignant glioma tissue and in the tumor periphery by infiltrative tumor tissue. Typical histopathological characteristics include the presence of nuclear atypia, mitotic activity, microvascular proliferation and/or necrosis. Predominantly, this tumor type affects adult patients between 45 and 75 years of age and men more often as well as women with ethnic predisposition [91].

Four gene expression based molecular subtypes of GBM, termed proneural, neural, classic and mesenchymal, have been distinguished by high-throughput technologies [93]. Several recent high-throughput studies revealed now different biological subgroups of GBM characterized by distinct DNA methylation profiles and associated gene mutations [94,95]. In contrast to molecular classification based on gene expression, classifications based on methylation profiles are probably more robust as it remains stable over tumor evolution and likely reflects the cell of origin. The methylation profile subgroups are related to MGMT promoter methylation, tumor location and patient age and outcome (Table 1).

### 3.3. Pathogenesis

In most patients, GBM develop de novo without any previous lesion as so-called primary GBM. WHO grade II and III gliomas might show transformation into secondary GBM. Both clinical and molecular data support the hypothesis that gliomas may develop from the mutation of different genes but affect the same cellular pathways. Key molecular features of GBM were identified including genetic abnormalities in receptor tyrosine kinase (RTK) genes that dysregulate growth factor signaling, activation of the phosphatidylinositol-3-OH kinase (PI3K) pathway and inactivation of the p53 and retinoblastoma tumor suppressor pathways [96]. Primary and secondary GBM represent distinct disease entities that affect different age groups of patients and develop through distinct genetic aberrations [94,97]. In detail, genetic aberrations occur in primary GBM with the following frequencies: 10–18% loss of neurofibromatosis 1 (NF1) gene, 15–25% mutation of PI3K, 3–60% abnormalities in RTK genes and 60–80% activating TERT promoter mutation. In contrast, secondary GBM feature with 60–80% mutation of isocitrate dehydrogenase 1 or 2 (IDH) genes, 15% abnormalities in RTK genes and 57% mutation or loss of ATRX gene. In particular, TERT and ATRX mutations are mutually exclusive in gliomas, suggesting either mutation in one of the major genes associated with TA or ALT as TMM may be sufficient to avoid replicative senescence by telomere loss and to drive glioma formation (Table 1).

### 3.4. Biomarkers and Current Treatment

Despite multimodal treatment including neurosurgical resection or biopsy, chemo-and/or radiotherapy WHO grade IV gliomas still have a very poor prognosis with a median overall survival of 14.6 months [92,98,99]. The growing knowledge about the molecular basis of this cancer in the past years shifted therapeutic interventions from primary empiric to molecular targeted ones, but such therapies failed in phase III trials and the classic multimodal treatment modalities remain the mainstay of therapy [94,95,100]. Thus, the outcome of patients with GBM has to date not significantly changed and is very poor with median patient survival durations of approximately 14 to 17 months in clinical trials and around 12 months in population-based studies [95]. For WHO 2016 classification of gliomas the diagnostic biomarkers include IDH mutations, 1p/19q codeletion, histone-H3-encoding genes H3F3A or HIST1H3B/C K27M (H3-K27M) mutations. Beside predictive glioma biomarkers in clinical use, such as 6-*O*-methylguanine-DNA methyltransferase (MGMT) promoter methylation and 1p/19q codeletion, further novel predictive biomarkers emerge for guiding the post-surgery treatment of patients, e.g., BRAF and IDH mutations, EGFR amplification and vIII variant expression, FGFR-TACC fusion. Additional prognostic roles of TERT and ATRX were recently demonstrated for adult infiltrating gliomas with WHO 2016 diagnosis [101].

In addition to genetic and chromosomal aberrations, some of the diagnostic biomarkers report epigenetic aberrations as well [95] (Table 1). In detail, presence of IDH1 or IDH2 mutations causes neomorphic enzymatic activity that result in conversion of alpha-ketoglutarate to d-2-hydroxygluterate, which inhibits dioxygenases that depend on alpha-ketoglutarate, such as ten-eleven translocation (TET) family 5-methylcytosine hydroxylases and specific histone-lysine demethylases [102]. Inhibition of these enzymes eventually leads to glioma CpG island methylator phenotype (G-CIMP) with widespread hypermethylation of CpG islands and favorable clinical behavior in younger patients [103].

Another example is the MGMT promoter methylation that has been linked with benefit from therapy with the DNA alkylating agent temozolomide and prolonged survival of GBM patients [95]. The major temozolomide induced DNA adduct 6-*O*-methylguanine is effectively repaired by MGMT. Hypermethylation of an CpG island in the MGMT promoter results in transcriptional repression and in reduced MGMT expression. The 6-*O*-methylguanine repair capacity by MGMT is reduced and renders tumor cells more sensitive to temozolomide.

H3-K27M mutation leads to global reduction of histone-H3-lysine 27 (H3K27) trimethylation and is often found in pediatric diffuse gliomas with high potential of targeted therapy [94,95].

From identified biomarkers and the genes involved in pathogenesis of cancers including gliomas it becomes evident that a number of dysregulated genes are involved in epigenetic mechanisms. Thus, epigenetic beside genetic alterations are of clinical importance and potential therapeutic targets for gliomas.

## 4. Epigenetic Regulation of TMMs

Dysregulated genes of TMMs are associated with specific subcategories for diffuse glioma diagnosis as defined by distinct genetic and epigenetic profiles as outlined before. Strategies targeting both TMMs are clinically important to avoid drug resistance as some tumors including gliomas show evidence of both telomerase and ALT activity (reviewed in [36,104]). In this regard, epigenetic cancer therapy is promising and might be useful for targeting TMMs, especially ALT. In contrast to TA, the ALT process involves multiple genes for HR and DNA synthesis, which are vital for repair and replication of DNA in normal cells, and their usability as tumor targets is not clear.

### 4.1. Epigenetic Mechanisms Regulating TA

It is well documented that TA is regulated in addition to genetic by epigenetic mechanisms (reviewed in [3,34,105,106,107]) and epigenetic regulation of TA is associated with complex changes in lifestyle [108]. TA is controlled by multiple mechanisms [3], e.g., down regulation of DNMTs reduces the hypermethylated state of the hTERT promoter by allowing repressor binding, chromatin remodeling changes the state of histones at the hTERT promoter by influencing the binding of transcription factors, miRNAs can change in posttranscriptional way hTERT transcript directly or indirectly via affecting regulatory transcription factors, and dietary compounds can influence hTERT by modulation the activity of DNMTs and histone-modifying enzymes. Dietary compounds that effect hTERT in tumor cells include tea polyphenols (catechins), isoflavones/phytoestrogens (genistein), polyphenols (resveratrol), isothiocyanates (sulforaphane), and yellow pigments (curcumin) [3]. Epigenetic diet that selectively affects the epigenome of cancer cells to control TA appears to be promising for prevention and treatment of cancers and may enter in the future practice in medicine as clinical nutrition. However, evidence of the significance of lifestyle-induced effects on TA and glioma therapy needs to be evaluated.

The mechanisms behind these regulations of telomerase appear to be rather complex networks and not simple linear processes. For example, full-length LINE-1 elements (L1s, long interspersed nuclear elements-1) are repeat-rich intronic or intergenic (junk) sequences, and are transcribed as euchromatin RNAs (ecRNAs) that may play a role in chromosome structure and regulation by promoting a more open chromatin state [109]. L1s become inactivated early in development, while recent evidence indicates they can be activated in cancer [110,111], as well as neurodegeneration (reviewed in [112]) and during development in the brain (reviewed in [113,114]).

Other repeat-rich RNAs are TERRA transcripts that may regulate TA via multiple mechanisms. TERRAs are long non-coding RNAs that are transcribed from subtelomeric and telomeric DNA in telomeric repeats with inhibitory role on TA [32]. About 7% of TERRA transcripts are 3′ end polyadenylated but only unpolyadenylated TERRA transcripts remain associated with telomeric heterochromatin to play crucial protective functions important for telomere maintenance [115]. TERRA compete in vitro with the telomere substrate to block TA [67,116]. In detail, TERRA has a stronger affinity for telomerase RNA subunit hTR than for the 3’ end of telomere DNA overhangs as the telomeric substrate [117]. TERRA function in vivo can be prevented by heterogeneous nuclear ribonucleoprotein A1 (hnRNPA1) that is associated with TERRA and telomeres. It was suggested that TERRA inhibition of TA in vivo depend on local concentrations of telomerase, hnRNPA1, TERRA and telomeric substrate and that TA needs balanced equal concentrations of both inhibitors hnRNPA1 and TERRA to act on the telomeric substrate [118]. It is not clear if TERRA act as positive or negative regulators of TA [115]. We recently developed tumor cell lines with elevated expression of recombinant TERRA telomeric repeats and demonstrated partial inhibition of TA without growth inhibition [119]. Such cell line models may be used to better understand the multiple roles of TERRA in the cell, but also for the functions not related to telomere biology in which a cell free form of TERRA enriched in extracellular exosomes can stimulate inflammatory cytokines in immune responsive cells [120].

TERRA levels are regulated by telomere length through EMs via increased trimethylation of telomeric H3K9 and HP1α [121]. During the cell cycle TERRA declines in late S phase before increasing again to ensure renewal of the TERRA pool [122]. Transcriptional regulation of TERRA is not well studied, but includes chromatin remodelers ATRX and CTCF, cohesin subunit Rad21, nuclear respiratory factor 1 (NRF1) and peroxisome proliferator-activated receptor gamma coactivator 1-alpha (PGC-1α) [123,124,125,126]. NRF1 and PGC-1α are regulated upstream by adenosine 5′-monophosphate (AMP)-activated protein kinase (AMPK). Stimulation of AMPK in cancer cells and in non-proliferating normal cells increased TERRA levels and links metabolism to telomere fitness important for cancer and aging [124]. It is speculated that TERRA may be part of an antioxidant response against excessive reactive oxygen species (ROS) at telomeres, as the high content of guanine residues may be prone to oxidation and thus shield the telomeric repeats from ROS, and the TERRA transcription process may positively affect telomeres by chromatin remodeling or telomeric loop formation. AMPK/PGC-1α activation mechanisms include type 2 diabetes drug metformin, life style factors, e.g., endurance exercise and dietary restriction, but also sirtuin activators as EMs such as resveratrol, that all show translational potential for TMM intervention.

### 4.2. Epigenetic Mechanisms Regulating ALT

**Figure 1:**
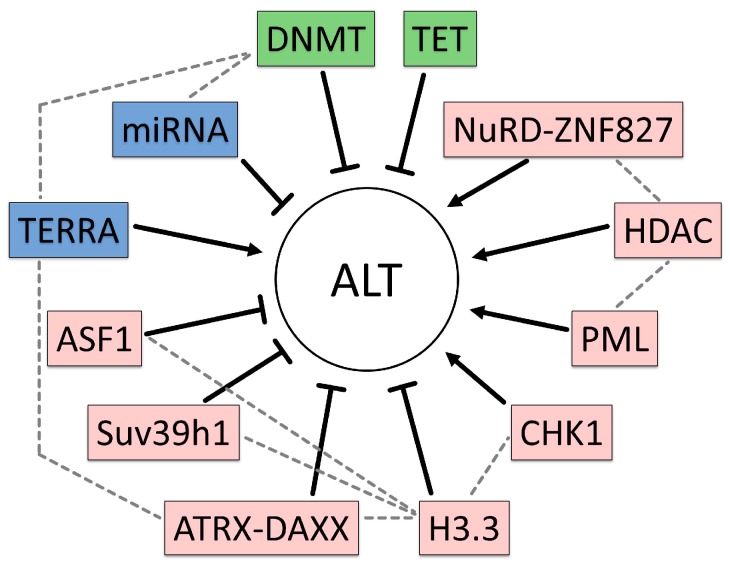
Schematic overview of EMs regulating ALT and their potential interplay. ALT activity is regulated via a complex network of epigenetic modifiers. The effects are marked with arrows or inhibitory arrows in black. EM marked by colors: DNA (green), RNA (blue) and histone (red). In addition to influencing ALT activity they also interact with each other (grey dotted lines).

We searched for preclinical evidence in the published literature that ALT is directly regulated by EMs and results are listed in Table 2. Preclinical data from mouse models and human in vitro studies demonstrate that different EMs regulate ALT and that such mechanisms might be critical for targeting human cancer in vivo (Figure 1).

DNA hypomethylation at chromosome ends in Dnmt-deficient and TA-deficient Tr (–/–) mouse models was accompanied with ALT phenotype detected by increased frequency of telomere recombination [19,127]. Dicer1 knock-out mouse model showed a miR-290 dependent pathway that targets Rbl2, a transcriptional repressor of DNA methyltransferases Dnmt3a and Dnmt3b expression, controlling DNA methylation and telomere recombination [128]. In contrast, hypomethylation of subtelomeric DNA in human tumor cells was not required for telomere recombination, although reduced chromatin compaction is often coincident with ALT [130]. Hypomethylation of human fibroblast cells isolated from patients with DNMT3b mutations was associated with abnormally short telomeres and increased levels of TERRA transcripts, but no increase of telomere recombination [131]. Low methylation levels at several CpG rich subtelomeric TERRA promoters correlate to upregulation of TERRA transcripts and to elongated telomeres and low levels of ALT specific C-Circles in human placenta, but the mechanistic link is unknown [21]. TA cells in humans as in mice show denser methylation than normal cells, being almost completely methylated and exhibit reduced levels of TERRA [19,141]. TERRA levels in astrocytoma correlate with clinical grade, low or absent TA and the presence of ALT as determined by long telomere lengths [78], making TERRA transcripts a candidate as marker for favorable prognosis associated with GBM [142].

Regulation of ALT mechanism by miRNAs is compared to TA yet not well studied (reviewed in [3,43]). As mentioned before, evidence exists for regulation of ALT by the miR-290 dependent pathway that controls DNA methylation and telomere recombination [128]. Genes associated with HR and ALT, such as RAD50, BRCA1, NBS1 are reported to be linked to specific miRNAs miR-183, miR-24 and miR-629, respectively [143,144]. These miRNAs may play a role in regulation of ALT, but this needs to be demonstrated. Positive role of TERRA on ALT was evident after knock-out a major TERRA locus [132]. TERRA promoters in human were initially identified as located on subtelomere loci of around half of chromosome ends [145]. Only two of the TERRA loci corresponding to 20q and Xp subtelomeres were confirmed recently by double RNA-FISH to encode indeed true TERRA features of subtelomere and telomere sequences [132]. By using CRISPR-Cas9 technology, the major locus for human TERRA transcription was identified at 20q as deletion caused a dramatic decrease in TERRA levels, while deletion of the Xp locus did not result in decreased TERRA levels. Cancer cell lines with the 20q-TERRA deletion showed dramatic loss of telomere sequences and the induction of a massive DDR in cancer cell lines using either TA or ALT. For example, an essential role of TERRA for ALT has been suggested by forming RNA/DNA hybrids with the telomeric C-rich strand important for a permissive environment for telomere recombination (reviewed in [61,146]).

TA-deficient Tr (–/–) mouse cells show decreased H3K9 and H4K20 trimethylation in telomeric and subtelomeric chromatin, as well as increased H3 and H4 acetylation at these regions and T-SCE associated with ALT activity [127]. Study of a leukemia mouse model revealed histone methyltransferase Suv39h1-deficient hematopoietic cells that showed less critical short telomeres and upregulated genes involved in ALT mechanism [133]. Suv39h1 mediates heterochromatin formation in pericentric and telomeric regions by trimethylation of lysine 9 of histone 3 (H3K9me3). Activation of ALT in mouse cells with dysfunctional chromatin is in line with the observation in human isogenic fibroblast cell models and cancer cell lines that chromatin of telomeres from ALT as compared to TA is more relaxed by lower occupancy of H3 and H3K9me3 and associated with increased TERRA expression [126]. Histone chaperone anti-silencing factor 1 (ASF1) assists the transfer of H3.1-H4 histone dimers to chromatin assembly factor 1 (CAF-1) or H3.3-H4 histone dimers to histone regulator A (HIRA) for nucleosome assembly [147] and disruptions of ASF1 cause histone management dysfunction and ALT [57]. Co-depletion of ASF1 paralogs ASF1a and ASF1b induced in both primary and cancer cells the manifestation of ALT hallmarks and repression of hTERT transcription and diminished TA. Both TMMs are repressed by each other in the cell due to chromatin remodeling and DNA methylation [148,149]. TA is repressed in ALT cells by several EMs, such as hypermethylation of the hTR promoter and histone H3 and H4 hypoacetylation as well as H3K9 methylation of both the hTERT and hTR promoter. Reason of the suppression is not clear, but genome instability observed in ALT may play a role [150]. Ectopic expression in ALT cells of both hTERT and hTR was shown to exert TA-related canonical and noncanonical functions that affect tumor genome evolution through suppression or induction of polyploidization by whole-genome duplication [150]. Next generation sequencing displayed variant repeats that were interspersed throughout the telomeres of ALT cells and recruit nuclear receptors [151]. Orphan nuclear receptors of the NR2C/F types bind to the predominant c-type variant (TCAGGG) repeats at ALT telomeres and bridge with other target loci to drive local telomere DNA addition by targeted telomere insertion (TTI) mechanism that may contribute to the complex karyotype in ALT [152]. Histone chaperone ATRX represses ALT [56] and this suppression is dependent on the histone chaperone DAXX [134]. It was suggested that loss of the chromatin remodeling factor ATRX promotes defective telomere chromatin with the persistence of aberrant DNA secondary structures, which in turn present a barrier to DNA replication, leading to replication fork stalling, collapse, HR and subsequent recombination-mediated telomere synthesis in ALT. These preclinical results are supported by exome sequencing of pediatric and young adult GBMs that demonstrated strong association between the presence of recurrent mutations in H3F3A/ATRX/DAXX/TP53 and ALT [135]. H3F3A encodes histone H3.3 and H3.3 phosphorylation by CHK1 is important for chromatin maintenance in human ALT cancer cells as inhibition of serine/threonine kinase CHK1 leads to reduced levels of H3.3 serine 31 phosphorylation and reduced cell viability [136]. The complex NuRD–ZNF827 is recruited to telomeres by nuclear receptors TR4 and COUP-TF2 encoded by NR2C2 and NR2F2, respectively [137]. The NuRD complex contains multiple subunits, including the histone deacetylase core proteins HDAC1 and HDAC2. The NuRD chromatin remodeling complex together with the zinc-finger protein ZNF827 lead in ALT cells to decreased shelterin binding, hypoacetylation of telomeric chromatin, enhanced telomere-telomere interactions and recruitment of HR proteins, creating an environment that promotes recombination and ALT activity. PML as component of the APBs induces clustering and compaction of colocalized telomere repeats, shelterin TRF2 depletion and DDR at telomeres and promotes ALT by changing the telomeric chromatin state [138]. Histone deacetylase HDAC9 regulates ALT activity by formation of APBs and C-circles in ALT positive cells and upon depletion in ALT cell lines the replicative capacity were compromised [139]. Histone deacetylase HDAC5 is recruited to long telomeres in ALT and TA cancer cell lines and ensures maintenance of telomeres whereas depletion of HDAC5 lead to short and homogeneous telomere length and sensitization to chemotherapy [140].

In conclusion, various epigenetic mechanisms regulate both TA and ALT through RNA, DNA and histone modification, but are often combined with genetic mechanisms such as gene mutations.

## 5. Epigenetic Therapy of Diffuse Gliomas

**Figure 2:**
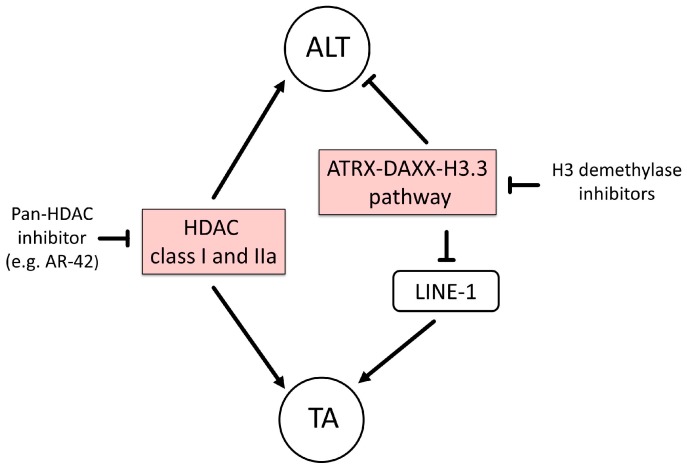
Schematic examples of potential epigenetic therapy targeting ALT and TA in glioma. Left: Inhibitory compounds against histone deacetylase HDAC class I and IIa may affect directly both ALT and TA. Right: The histone chaperones ATRX/DAXX deposit the H3.3 at telomeric chromatin and thereby maintain silencing histone marks such as H3K9me3 and H3K27me3. Demethylase inhibitors block demethylation of H3 with possible change in chromatin structure that may block directly ALT and TA via LINE-1. Symbols as defined in Figure 1.

TMM profiling may have clinical importance for diagnosis, prognosis and treatment decision of tumors as described before. The question of how the profiling will be carried out best in the clinic has to be considered in the context of clinical samples studied and of specificity/sensitivity of available TMM assays. Furthermore, personalized treatment decisions based on TMM status that can be directly targeted for clinical purpose are challenging and not established in therapy of cancers (reviewed in [36]). Targeting approaches of TA e.g., by telomerase oligonucleotide inhibitor GRN163L in clinical studies shows limitations and challenges (reviewed in [153,154]) and the recent success in deciphering the ALT mechanisms provides multiple essential steps for potential targeting the ALT mechanism (reviewed in [40]). It becomes evident from the studies already described that ALT is based on dysregulation of telomere chromatin by remodeling as was speculated (reviewed in [155]) and multiple EMs in combination with genetic mechanisms affects ALT (Figure 1). These mechanisms are potential drug targets with some of them acting on one or the other TMM (Figure 2). Targeting ALT and TA in combination may be relevant to avoid tumor resistance by switching to another TMM [104].

Strategies that modulate methylation at telomeres in ALT are expected to hamper ALT cells (Figure 1). However, direct restoration of DNMTs by activators is not studied and inhibition of DNA demethylation by TET enzymes does not seem promising for targeting ALT as TET product levels 5-Hydroxymethylcytosine (5hmC) are low in brain tumors (reviewed in [156]). 5hmC is generated from 5-Methylcytosine (5mC) by the action of the TET enzymes and may be an intermediate to further oxidation and finally demethylation of 5mC. 5hmC was decreased to 1–2% in gliomas compared with 62% in normal brain [157]. Furthermore, triple knockout mouse models for all three TET proteins showed a role of TETs in DNA demethylation and repression of ALT [129].

Over the last decade, epigenetic therapy by histone deacetylase (HDAC) inhibitors was demonstrated by several preclinical and clinical studies and may become promising for GBM therapy (reviewed in [158,159]). Some HDAC inhibitors are approved epigenetic drugs that effectively radiosensitize various tumors, including GBM. Mechanistically, HDAC inhibitors seem to prevent DNA DSB repair, but show multiple anti-cancer mechanisms that inhibit oncogenes and tumor angiogenesis, and upregulate expression of tumor suppressor genes and the immune system. HDAC inhibitors are currently being tested in clinical trials on GBM as either monotherapy or combination therapy (reviewed in [158]). In contrast, no drugs that target histone methylation or epigenetic readers are under clinical study, although targeting methyltransferases and demethylases are expected to have high potential. HDAC5 and HDAC9 that effect ALT belong to class IIa Zn^2+^-dependent HDACs and are tissue specifically expressed, including brain. HDAC9 is overexpressed in GBM patients with poor prognosis and promotes tumor formation of glioblastoma via activation of the TAZ-mediated EGFR pathway [160]. These preclinical results are promising, but specific inhibitors for HDAC9 are not available (reviewed in [158]). However, several HDAC inhibitors that are less specific but target class IIa and show good blood brain barrier penetration may be candidates for preclinical studies targeting ALT and TA (Figure 2). For example, the pan-HDAC inhibitor AR-42 (hydroxamate derivative) inhibits TA in a PTEN-null glioma cell line via Akt-dependent mechanism [161]. The NuRD–ZNF827 complex contains HDAC1 and HDAC2 that are Zn^2+^-dependent class I HDACs (Figure 1 and Figure 2). Targeting HDAC1, HDAC2 or pan-HDACs with various HDAC inhibitor classes of short-chain fatty acids, cyclic benzamides, hydroxamate derivates and Diallyl-trisulfides (DATS) might be a rational for targeting ALT in gliomas (reviewed in [159]).

Recent preclinical studies suggest a potential of targeted therapy against H3.3 mutant gliomas (reviewed in [94,95]). GSK-J4 inhibitor which inhibits specific K27 demethylase increased H3K27 trimethylation and showed potent antitumor activity against H3-K27M mutant glioma cells [162]. The multi-histone deacetylase inhibitor panobinostat showed promising synergistic activity with GSK-J4 [163]. Further histone modifications often disrupted by genetic alterations in pediatric and adult gliomas may be targeted (reviewed in [164,165,166,167]).

Such strategies of epigenetic targeting the histone code seem to be applicable for reactivation of the dysfunctional ATRX/DAXX/H3.3 pathway in tumors with ALT but this remains to be demonstrated. The ATRX/DAXX/H3.3 pathway is also important for heterochromatin silencing including LINE-1 elements (reviewed in [50]). Targeting this pathway may affect both ALT and TA cancers important to avoid resistance [104]. L1s appear to be a target in the rational treatment of TA cancers, as telomerase-positive tumor cells critically depend on expression of active L1s for telomere maintenance [110]. On the one hand, restoring the ATRX/DAXX/H3.3 pathway activity in ALT tumors represses ALT activity as described before, and, on the other hand, activating the pathway to maintain the H3K9me3 modification or blockage of specific demethylases/methyltransferases in TA tumors may reduce L1s activation capacity of TA (Figure 2).

The specific role of ncRNAs on TMMs has to be deciphered by further preclinical studies. Targeting ncRNAs, such as TERRA or specific ecRNA and miRNA, by future RNA-based therapeutics that may block ALT and TA in tumors is a challenging outlook and promise a big impact [168]. Functional studies have revealed several microRNA therapeutics: on the one hand, miRNA mimics; and, on the other hand, molecules targeted at miRNAs (antimiRs) that have reached clinical development [169]. HDAC and miRNAs have been shown to interact with each other (reviewed in [170]), combination of selective HDAC inhibitors and new miRNA silencing techniques (reviewed in [171,172]) may form a new perspective in epigenetics-based therapies targeting TMMs in gliomas.

## 6. Conclusions

Prognosis of GBM has not improved significantly, although diagnostic and therapeutic strategies have advanced [173]. Explanations might be the heterogeneity of GBM tumors and the common multimodal treatment strategies with limitations in terms of efficiency and side effects. Classification of GBM patients based on genetic, epigenetic and transcription profiling may allow implementing the personalized medicine concept for individual drug treatment decisions and predicting patient outcome. Important progress from basic science on deciphering details of TMMs highlight associations of TA and ALT with closed and open chromatin structures at telomeres, respectively, that may be targeted by epigenetic therapy. TMM profile markers predict outcomes in GBM patients and are characterized for TA and ALT by mutations in the hTERT promoter and in genes important for telomeric chromatin remodeling, respectively. Recent findings from preclinical science and first clinical data indicate the prognostic importance of mutations affecting the histone code and the telomere length in adult and pediatric diffuse gliomas that may be targets for novel epigenetic therapeutics [164]. Coincidence with recent discoveries of the ALT mechanism will allow overcoming the limitations of available preclinical TMM models for glioma that are important for validation of novel therapeutic strategies to become effective in clinical practice.

Based on preclinical and clinical information available, we propose further work with a translational approach to evaluate the significance of epigenetic therapeutics for targeting telomere maintenance in gliomas. In detail, preclinical studies may allow validating of most promising candidates for clinical studies, such as histone modifying enzyme inhibitors and RNA targeting strategies, also in combination with current therapeutic concepts. Future studies may prove therapeutic effects on non-malignant cells as ALT was identified in pluripotent cells and DNA methylation may have important functions in brain. Epigenetic targeting strategies of TMMs in glioma may also be applicable to other cancers, as ALT is anticipated as anti-TA resistance mechanism.

## Figures and Tables

**Table 1:** Genetic aberrations connected to epigenetic mechanism (EM) and their occurrence in molecular subgroups of glioblastoma multiforme (GBM).

Genetic Aberrations ^1^	Molecular Subgroups of GBM						
	PXA-Like	LGG-Like	H3K27	H3G34	IDH-Mutant	RTK I	RTK II
TERT promoter mutation						X	X
ATRX mutation			X	X	X		
H3K27 mutation			X				
H3.3 G34 mutation				X			
TP53 mutation			X	X	X		
IDH1/2 mutation					X		
G-CIMP					X		
DNA hypomethylation				X			
Patients Characteristics							
MGMT promoter methylation per group (%)	~15	20–25	<5	~75	~90	~40	~40
Age (years)	<18	<18	<18	<30	20–50	20–50	>50
Median OS (months)	>36	>36	~12	~24	~30	~12–14	~12–14

^1^ Only genetic aberrations from [95] connected to EM were included in the table, the full information on the genetic and epigenetic profiles of the subgroups can be found in Figure 3 of [95]. The genetic aberration can be found in the subgroup if marked with an X. G-CIMP, glioma-associated CpG-island methylator phenotype; OS, overall survival; PXA, pleomorphic xanthoastrocytoma; LGG, low-grade glioma; RTK, Receptor tyrosine kinase.

**Table 2:** Evidence of epigenetic mechanisms effecting ALT in human or mouse.

EM ^1^	Effect ^2^	Modifier	Target	Mode of Action	References
DNA	−	Dnmt1, Dnmt3a/b ^4^	Subtelomere	Methylation	[19]
	−	TA, Tr ^4^	Subtelomere	Methylation	[127]
	−	Rbl2-dependent Dnmt3a/b ^4^	Subtelomere	Methylation	[128]
	−	Tet	CpG	Demethylation	[129]
	+	?	CpG rich TERRA promoter	Demethylation	[21]
	No	DNMT3b	Subtelomere	Methylation	[130,131]
RNA	−	miR-290, Dicer1 ^4^	Rbl2-dependent DNMT3a/b	Upregulation	[128]
	+	20q-TERRA	Telomeres ^3^	RNA/DNA hybrids ^3^	[132]
Histone	−	TA, Tr ^4^	Telomeric, subtelomeric chromatin	Trimethylation (H3K9, H4K20), deacetylation (H3, H4)	[127]
	−	Suv39h1 ^4^	Telomeric chromatin	Trimethylation (H3K9)	[133]
	?	?	Telomeric, subtelomeric chromatin	Low levels of H3, H3K9me3	[126]
	−	ASF1a/b	Chromatin ^3^	H3.1 and H3.3 loading ^3^	[57]
	−	ATRX	Chromatin ^3^	?	[56]
	−	ATRX-DAXX	Telomeric chromatin	H3.3 loading	[134,135]
	−	?	H3.3	Methylation, acetylation ^5^	[135]
	+	CHK1	H3.3S31	Phosphorylation	[136]
	+	NuRD-ZNF827	Telomeric chromatin	Deacetylation, decreased shelterin binding	[137]
	+	PML	Telomeric chromatin	Compaction, TRF2 depletion	[138]
	+	HDAC9	PML	?	[139]
	+	HDAC5	Telomeric chromatin	?	[140]

^1^ Epigenetic mechanisms (EM) based on DNA, non-coding RNA or histone modifications; ^2^ Activating (+), inhibiting (−), neutral (No) or unknown (?) effect on ALT; ^3^ Suggested target or mode of action; ^4^ Data from mouse models; ^5^ Mutations K27M and G34R/V in the gene of H3.3 change the epigenetic modifications of the histone tail, which are strongly associated with ALT.

## References

[1] Biswas S., Rao C.M. (2017). Epigenetics in cancer: Fundamentals and beyond. Pharmacol. Ther..

[2] You J.S., Jones P.A. (2012). Cancer genetics and epigenetics: Two sides of the same coin?. Cancer Cell.

[3] Lewis K.A., Tollefsbol T.O. (2016). Regulation of the telomerase reverse transcriptase subunit through epigenetic mechanisms. Front. Genet..

[4] Blasco M.A. (2007). The epigenetic regulation of mammalian telomeres. Nat. Rev..

[5] Arnoult N., Karlseder J. (2015). Complex interactions between the DNA-damage response and mammalian telomeres. Nat. Struct. Mol. Biol..

[6] De Lange T. (2005). Shelterin: The protein complex that shapes and safeguards human telomeres. Genes Dev..

[7] Erdel F., Kratz K., Willcox S., Griffith J.D., Greene E.C., de Lange T. (2017). Telomere recognition and assembly mechanism of mammalian shelterin. Cell Rep..

[8] Hayflick L., Moorhead P.S. (1961). The serial cultivation of human diploid cell strains. Exp. Cell Res..

[9] Shay J.W., Wright W.E. (2000). Hayflick, his limit, and cellular ageing. Nat. Rev. Mol. Cell Biol..

[10] Howard B.H. (1996). Replicative senescence: Considerations relating to the stability of heterochromatin domains. Exp. Gerontol..

[11] Matsumura T., Malik F., Holliday R. (1989). Levels of DNA methylation in diploid and SV40 transformed human fibroblasts. Exp. Gerontol..

[12] Reddel R.R. (2010). Senescence: An antiviral defense that is tumor suppressive?. Carcinogenesis.

[13] Sharpless N.E., DePinho R.A. (2007). How stem cells age and why this makes us grow old. Nat. Rev. Mol. Cell Biol..

[14] Aubert G. (2014). Telomere dynamics and aging. Prog. Mol. Biol. Transl. Sci..

[15] Zeng S., Liu L., Sun Y., Xie P., Hu L., Yuan D., Chen D., Ouyang Q., Lin G., Lu G. (2014). Telomerase-mediated telomere elongation from human blastocysts to embryonic stem cells. J. Cell Sci..

[16] Rivera T., Haggblom C., Cosconati S., Karlseder J. (2017). A balance between elongation and trimming regulates telomere stability in stem cells. Nat. Struct. Mol. Biol..

[17] Liu L., Bailey S.M., Okuka M., Munoz P., Li C., Zhou L., Wu C., Czerwiec E., Sandler L., Seyfang A. (2007). Telomere lengthening early in development. Nat. Cell Biol..

[18] Neumann A.A., Watson C.M., Noble J.R., Pickett H.A., Tam P.P., Reddel R.R. (2013). Alternative lengthening of telomeres in normal mammalian somatic cells. Genes Dev..

[19] Gonzalo S., Jaco I., Fraga M.F., Chen T., Li E., Esteller M., Blasco M.A. (2006). DNA methyltransferases control telomere length and telomere recombination in mammalian cells. Nat. Cell Biol..

[20] Slatter T.L., Tan X., Yuen Y.C., Gunningham S., Ma S.S., Daly E., Packer S., Devenish C., Royds J.A., Hung N.A. (2012). The alternative lengthening of telomeres pathway may operate in non-neoplastic human cells. J. Pathol..

[21] Novakovic B., Napier C.E., Vryer R., Dimitriadis E., Manuelpillai U., Sharkey A., Craig J.M., Reddel R.R., Saffery R. (2016). DNA methylation mediated up-regulation of terra non-coding RNA is coincident with elongated telomeres in the human placenta. Mol. Hum. Reprod..

[22] Pickett H.A., Reddel R.R. (2015). Molecular mechanisms of activity and derepression of alternative lengthening of telomeres. Nat. Struct. Mol. Biol..

[23] Colgin L.M., Reddel R.R. (1999). Telomere maintenance mechanisms and cellular immortalization. Curr. Opin. Genet. Dev..

[24] Hanahan D., Weinberg R.A. (2000). The hallmarks of cancer. Cell.

[25] Hanahan D., Weinberg R.A. (2011). Hallmarks of cancer: The next generation. Cell.

[26] Cesare A.J., Reddel R.R. (2010). Alternative lengthening of telomeres: Models, mechanisms and implications. Nat. Rev..

[27] Greider C.W., Blackburn E.H. (1985). Identification of a specific telomere terminal transferase activity in tetrahymena extracts. Cell.

[28] Cech T.R. (2004). Beginning to understand the end of the chromosome. Cell.

[29] Cong Y.S., Wright W.E., Shay J.W. (2002). Human telomerase and its regulation. Microbiol. Mol. Biol. Rev..

[30] Mergny J.L., Riou J.F., Mailliet P., Teulade-Fichou M.P., Gilson E. (2002). Natural and pharmacological regulation of telomerase. Nucleic Acids Res..

[31] Blackburn E.H., Greider C.W., Szostak J.W. (2006). Telomeres and telomerase: The path from maize, tetrahymena and yeast to human cancer and aging. Nat. Med..

[32] MacNeil D.E., Bensoussan H.J., Autexier C. (2016). Telomerase regulation from beginning to the end. Genes.

[33] Bell R.J., Rube H.T., Xavier-Magalhaes A., Costa B.M., Mancini A., Song J.S., Costello J.F. (2016). Understanding tert promoter mutations: A common path to immortality. Mol. Cancer Res..

[34] Koziel J.E., Fox M.J., Steding C.E., Sprouse A.A., Herbert B.S. (2011). Medical genetics and epigenetics of telomerase. J. Cell. Mol. Med..

[35] Cairney C.J., Keith W.N. (2008). Telomerase redefined: Integrated regulation of hTR and hTERT for telomere maintenance and telomerase activity. Biochimie.

[36] Reddel R.R. (2014). Telomere maintenance mechanisms in cancer: Clinical implications. Curr. Pharm. Des..

[37] Bryan T.M., Englezou A., Dalla-Pozza L., Dunham M.A., Reddel R.R. (1997). Evidence for an alternative mechanism for maintaining telomere length in human tumors and tumor-derived cell lines. Nat. Med..

[38] Bryan T.M., Englezou A., Gupta J., Bacchetti S., Reddel R.R. (1995). Telomere elongation in immortal human cells without detectable telomerase activity. EMBO J..

[39] Reddel R.R., Bryan T.M., Murnane J.P. (1997). Immortalized cells with no detectable telomerase activity. A review. Biochemistry.

[40] Dilley R.L., Greenberg R.A. (2015). Alternative telomere maintenance and cancer. Trends Cancer.

[41] Henson J.D., Reddel R.R. (2010). Assaying and investigating alternative lengthening of telomeres activity in human cells and cancers. FEBS Lett..

[42] Nittis T., Guittat L., Stewart S.A. (2008). Alternative lengthening of telomeres (ALT) and chromatin: Is there a connection?. Biochimie.

[43] Santambrogio F., Gandellini P., Cimino-Reale G., Zaffaroni N., Folini M. (2014). MicroRNA-dependent regulation of telomere maintenance mechanisms: A field as much unexplored as potentially promising. Curr. Pharm. Des..

[44] Henson J.D., Lau L.M., Koch S., Martin La Rotta N., Dagg R.A., Reddel R.R. (2017). The c-circle assay for alternative-lengthening-of-telomeres activity. Methods.

[45] Sakellariou D., Chiourea M., Raftopoulou C., Gagos S. (2013). Alternative lengthening of telomeres: Recurrent cytogenetic aberrations and chromosome stability under extreme telomere dysfunction. Neoplasia.

[46] Muntoni A., Reddel R.R. (2005). The first molecular details of alt in human tumor cells. Hum. Mol. Genet..

[47] Nabetani A., Ishikawa F. (2011). Alternative lengthening of telomeres pathway: Recombination-mediated telomere maintenance mechanism in human cells. J. Biochem..

[48] Ho A., Wilson F.R., Peragine S.L., Jeyanthan K., Mitchell T.R., Zhu X.D. (2016). Trf1 phosphorylation on t271 modulates telomerase-dependent telomere length maintenance as well as the formation of alt-associated pml bodies. Sci. Rep..

[49] Amorim J.P., Santos G., Vinagre J., Soares P. (2016). The role of ATRX in the alternative lengthening of telomeres (ALT) phenotype. Genes.

[50] Voon H.P., Wong L.H. (2016). New players in heterochromatin silencing: Histone variant H3.3 and the atrx/daxx chaperone. Nucleic Acids Res..

[51] Gocha A.R., Harris J., Groden J. (2013). Alternative mechanisms of telomere lengthening: Permissive mutations, DNA repair proteins and tumorigenic progression. Mutat. Res..

[52] O’Sullivan R.J., Almouzni G. (2014). Assembly of telomeric chromatin to create alternative endings. Trends Cell Biol..

[53] Flynn R.L., Cox K.E., Jeitany M., Wakimoto H., Bryll A.R., Ganem N.J., Bersani F., Pineda J.R., Suva M.L., Benes C.H. (2015). Alternative lengthening of telomeres renders cancer cells hypersensitive to atr inhibitors. Science.

[54] Lovejoy C.A., Li W., Reisenweber S., Thongthip S., Bruno J., de Lange T., De S., Petrini J.H., Sung P.A., Jasin M. (2012). Loss of ATRX, genome instability, and an altered DNA damage response are hallmarks of the alternative lengthening of telomeres pathway. PLoS Genet..

[55] Wong L.H., McGhie J.D., Sim M., Anderson M.A., Ahn S., Hannan R.D., George A.J., Morgan K.A., Mann J.R., Choo K.H. (2010). Atrx interacts with H3.3 in maintaining telomere structural integrity in pluripotent embryonic stem cells. Genome Res..

[56] Napier C.E., Huschtscha L.I., Harvey A., Bower K., Noble J.R., Hendrickson E.A., Reddel R.R. (2015). Atrx represses alternative lengthening of telomeres. Oncotarget.

[57] O′Sullivan R.J., Arnoult N., Lackner D.H., Oganesian L., Haggblom C., Corpet A., Almouzni G., Karlseder J. (2014). Rapid induction of alternative lengthening of telomeres by depletion of the histone chaperone asf1. Nat. Struct. Mol. Biol..

[58] Hu Y., Shi G., Zhang L., Li F., Jiang Y., Jiang S., Ma W., Zhao Y., Songyang Z., Huang J. (2016). Switch telomerase to ALT mechanism by inducing telomeric DNA damages and dysfunction of ATRX and DAXX. Sci. Rep..

[59] Koschmann C., Calinescu A.A., Nunez F.J., Mackay A., Fazal-Salom J., Thomas D., Mendez F., Kamran N., Dzaman M., Mulpuri L. (2016). ATRX loss promotes tumor growth and impairs nonhomologous end joining DNA repair in glioma. Sci. Transl. Med..

[60] Roumelioti F.M., Sotiriou S.K., Katsini V., Chiourea M., Halazonetis T.D., Gagos S. (2016). Alternative lengthening of human telomeres is a conservative DNA replication process with features of break-induced replication. EMBO Rep..

[61] Dilley R.L., Verma P., Cho N.W., Winters H.D., Wondisford A.R., Greenberg R.A. (2016). Break-induced telomere synthesis underlies alternative telomere maintenance. Nature.

[62] Min J., Wright W.E., Shay J.W. (2017). Alternative lengthening of telomeres can be maintained by preferential elongation of lagging strands. Nucleic Acids Res..

[63] Hung N.A., Eiholzer R.A., Kirs S., Zhou J., Ward-Hartstonge K., Wiles A.K., Frampton C.M., Taha A., Royds J.A., Slatter T.L. (2016). Telomere profiles and tumor-associated macrophages with different immune signatures affect prognosis in glioblastoma. Mod. Pathol..

[64] Kreilmeier T., Sampl S., Deloria A.J., Walter I., Reifinger M., Hauck M., Borst L.B., Holzmann K., Kleiter M. (2016). Alternative lengthening of telomeres does exist in various canine sarcomas. Mol. Carcinog..

[65] Royds J.A., Al Nadaf S., Wiles A.K., Chen Y.J., Ahn A., Shaw A., Bowie S., Lam F., Baguley B.C., Braithwaite A.W. (2011). The CDKN2A G500 allele is more frequent in gbm patients with no defined telomere maintenance mechanism tumors and is associated with poorer survival. PLoS ONE.

[66] Azzalin C.M., Reichenbach P., Khoriauli L., Giulotto E., Lingner J. (2007). Telomeric repeat containing RNA and RNA surveillance factors at mammalian chromosome ends. Science.

[67] Schoeftner S., Blasco M.A. (2008). Developmentally regulated transcription of mammalian telomeres by DNA-dependent RNA polymerase II. Nat. Cell Biol..

[68] Fuhrmann G., Jonsson F., Weil P.P., Postberg J., Lipps H.J. (2016). RNA-template dependent de novo telomere addition. RNA Biol..

[69] Hakin-Smith V., Jellinek D.A., Levy D., Carroll T., Teo M., Timperley W.R., McKay M.J., Reddel R.R., Royds J.A. (2003). Alternative lengthening of telomeres and survival in patients with glioblastoma multiforme. Lancet.

[70] Henson J.D., Hannay J.A., McCarthy S.W., Royds J.A., Yeager T.R., Robinson R.A., Wharton S.B., Jellinek D.A., Arbuckle S.M., Yoo J. (2005). A robust assay for alternative lengthening of telomeres in tumors shows the significance of alternative lengthening of telomeres in sarcomas and astrocytomas. Clin. Cancer Res..

[71] McDonald K.L., McDonnell J., Muntoni A., Henson J.D., Hegi M.E., von Deimling A., Wheeler H.R., Cook R.J., Biggs M.T., Little N.S. (2010). Presence of alternative lengthening of telomeres mechanism in patients with glioblastoma identifies a less aggressive tumor type with longer survival. J. Neuropathol. Exp. Neurol..

[72] Dorris K., Sobo M., Onar-Thomas A., Panditharatna E., Stevenson C.B., Gardner S.L., Dewire M.D., Pierson C.R., Olshefski R., Rempel S.A. (2014). Prognostic significance of telomere maintenance mechanisms in pediatric high-grade gliomas. J. Neuro-Oncol..

[73] Martinez P., Blasco M.A. (2011). Telomeric and extra-telomeric roles for telomerase and the telomere-binding proteins. Nat. Rev. Cancer.

[74] Low K.C., Tergaonkar V. (2013). Telomerase: Central regulator of all of the hallmarks of cancer. Trends Biochem. Sci..

[75] Heaphy C.M., de Wilde R.F., Jiao Y., Klein A.P., Edil B.H., Shi C., Bettegowda C., Rodriguez F.J., Eberhart C.G., Hebbar S. (2011). Altered telomeres in tumors with atrx and daxx mutations. Science.

[76] Marinoni I., Kurrer A.S., Vassella E., Dettmer M., Rudolph T., Banz V., Hunger F., Pasquinelli S., Speel E.J., Perren A. (2014). Loss of DAXX and ATRX are associated with chromosome instability and reduced survival of patients with pancreatic neuroendocrine tumors. Gastroenterology.

[77] Scarpa A., Chang D.K., Nones K., Corbo V., Patch A.M., Bailey P., Lawlor R.T., Johns A.L., Miller D.K., Mafficini A. (2017). Whole-genome landscape of pancreatic neuroendocrine tumours. Nature.

[78] Sampl S., Pramhas S., Stern C., Preusser M., Marosi C., Holzmann K. (2012). Expression of telomeres in astrocytoma who grade 2 to 4: Terra level correlates with telomere length, telomerase activity, and advanced clinical grade. Transl. Oncol..

[79] Naderlinger E., Holzmann K. (2017).

[80] Silvestre D.C., Pineda J.R., Hoffschir F., Studler J.M., Mouthon M.A., Pflumio F., Junier M.P., Chneiweiss H., Boussin F.D. (2011). Alternative lengthening of telomeres in human glioma stem cells. Stem Cells.

[81] Jeitany M., Pineda J.R., Liu Q., Porreca R.M., Hoffschir F., Desmaze C., Silvestre D.C., Mailliet P., Junier M.P., Londono-Vallejo A. (2015). A preclinical mouse model of glioma with an alternative mechanism of telomere maintenance (ALT). Int. J. Cancer.

[82] Borodovsky A., Meeker A.K., Kirkness E.F., Zhao Q., Eberhart C.G., Gallia G.L., Riggins G.J. (2015). A model of a patient-derived idh1 mutant anaplastic astrocytoma with alternative lengthening of telomeres. J. Neuro-Oncol..

[83] Sampl S., Holzmann K. (2017).

[84] Fuller G.N. (2008). The WHO Classification of Tumours of the Central Nervous System.

[85] Louis D.N., Ohgaki H., Wiestler O.D., Cavenee W.K., Burger P.C., Jouvet A., Scheithauer B.W., Kleihues P. (2007). The 2007 WHO classification of tumours of the central nervous system. Acta Neuropathol..

[86] Wohrer A., Waldhor T., Heinzl H., Hackl M., Feichtinger J., Gruber-Mosenbacher U., Kiefer A., Maier H., Motz R., Reiner-Concin A. (2009). The austrian brain tumour registry: A cooperative way to establish a population-based brain tumour registry. J. Neuro-Oncol..

[87] Louis D.N., Perry A., Reifenberger G., von Deimling A., Figarella-Branger D., Cavenee W.K., Ohgaki H., Wiestler O.D., Kleihues P., Ellison D.W. (2016). The 2016 world health organization classification of tumors of the central nervous system: A summary. Acta Neuropathol..

[88] Dolecek T.A., Propp J.M., Stroup N.E., Kruchko C. (2012). Cbtrus statistical report: Primary brain and central nervous system tumors diagnosed in the united states in 2005–2009. Neuro-Oncology.

[89] Siebzehnrubl F.A., Reynolds B.A., Vescovi A., Steindler D.A., Deleyrolle L.P. (2011). The origins of glioma: E pluribus unum?. Glia.

[90] Zong H., Parada L.F., Baker S.J. (2015). Cell of origin for malignant gliomas and its implication in therapeutic development. Cold Spring Harbor Perspect. Biol..

[91] Thakkar J.P., Dolecek T.A., Horbinski C., Ostrom Q.T., Lightner D.D., Barnholtz-Sloan J.S., Villano J.L. (2014). Epidemiologic and molecular prognostic review of glioblastoma. Cancer Epidemiol. Biomark. Prev..

[92] Wen P.Y., Kesari S. (2008). Malignant gliomas in adults. N. Engl. J. Med..

[93] Verhaak R.G., Hoadley K.A., Purdom E., Wang V., Qi Y., Wilkerson M.D., Miller C.R., Ding L., Golub T., Mesirov J.P. (2010). Integrated genomic analysis identifies clinically relevant subtypes of glioblastoma characterized by abnormalities in PDGFRa, IDH1, EGFR, and NF1. Cancer Cell.

[94] Masui K., Mischel P.S., Reifenberger G. (2016). Molecular classification of gliomas. Handb. Clin. Neurol..

[95] Reifenberger G., Wirsching H.G., Knobbe-Thomsen C.B., Weller M. (2016). Advances in the molecular genetics of gliomas-implications for classification and therapy. Nat. Rev. Clin. Oncol..

[96] Cancer Genome Atlas Research N. (2008). Comprehensive genomic characterization defines human glioblastoma genes and core pathways. Nature.

[97] Li Q.J., Cai J.Q., Liu C.Y. (2016). Evolving molecular genetics of glioblastoma. Chin. Med. J..

[98] Preusser M., de Ribaupierre S., Wohrer A., Erridge S.C., Hegi M., Weller M., Stupp R. (2011). Current concepts and management of glioblastoma. Ann. Neurol..

[99] Stupp R., Mason W.P., van den Bent M.J., Weller M., Fisher B., Taphoorn M.J., Belanger K., Brandes A.A., Marosi C., Bogdahn U. (2005). Radiotherapy plus concomitant and adjuvant temozolomide for glioblastoma. N. Engl. J. Med..

[100] Weller M., Wick W., Aldape K., Brada M., Berger M., Pfister S.M., Nishikawa R., Rosenthal M., Wen P.Y., Stupp R. (2015). Glioma. Nat. Rev. Dis. Prim..

[101] Pekmezci M., Rice T., Molinaro A.M., Walsh K.M., Decker P.A., Hansen H., Sicotte H., Kollmeyer T.M., McCoy L.S., Sarkar G. (2017). Adult infiltrating gliomas with WHO 2016 integrated diagnosis: Additional prognostic roles of ATRX and TERT. Acta Neuropathol..

[102] Xu W., Yang H., Liu Y., Yang Y., Wang P., Kim S.H., Ito S., Yang C., Wang P., Xiao M.T. (2011). Oncometabolite 2-hydroxyglutarate is a competitive inhibitor of alpha-ketoglutarate-dependent dioxygenases. Cancer Cell.

[103] Noushmehr H., Weisenberger D.J., Diefes K., Phillips H.S., Pujara K., Berman B.P., Pan F., Pelloski C.E., Sulman E.P., Bhat K.P. (2010). Identification of a CPG island methylator phenotype that defines a distinct subgroup of glioma. Cancer Cell.

[104] Shay J.W., Reddel R.R., Wright W.E. (2012). Cancer. Cancer and telomeres-an alternative to telomerase. Science.

[105] Zhu J., Zhao Y., Wang S. (2010). Chromatin and epigenetic regulation of the telomerase reverse transcriptase gene. Protein Cell.

[106] Lai S.R., Phipps S.M., Liu L., Andrews L.G., Tollefsbol T.O. (2005). Epigenetic control of telomerase and modes of telomere maintenance in aging and abnormal systems. Front. Biosci..

[107] Liu L., Lai S., Andrews L.G., Tollefsbol T.O. (2004). Genetic and epigenetic modulation of telomerase activity in development and disease. Gene.

[108] Falus A., Marton I., Borbenyi E., Tahy A., Karadi P., Aradi J., Stauder A., Kopp M. (2011). A challenging epigenetic message: Telomerase activity is associated with complex changes in lifestyle. Cell Biol. Int..

[109] Hall L.L., Lawrence J.B. (2016). RNA as a fundamental component of interphase chromosomes: Could repeats prove key?. Curr. Opin. Genet. Dev..

[110] Aschacher T., Wolf B., Enzmann F., Kienzl P., Messner B., Sampl S., Svoboda M., Mechtcheriakova D., Holzmann K., Bergmann M. (2016). Line-1 induces htert and ensures telomere maintenance in tumour cell lines. Oncogene.

[111] Shukla R., Upton K.R., Munoz-Lopez M., Gerhardt D.J., Fisher M.E., Nguyen T., Brennan P.M., Baillie J.K., Collino A., Ghisletti S. (2013). Endogenous retrotransposition activates oncogenic pathways in hepatocellular carcinoma. Cell.

[112] Szafranski K., Abraham K.J., Mekhail K. (2015). Non-coding rna in neural function, disease, and aging. Front. Genet..

[113] Erwin J.A., Marchetto M.C., Gage F.H. (2014). Mobile DNA elements in the generation of diversity and complexity in the brain. Nat. Rev. Neurosci..

[114] Richardson S.R., Morell S., Faulkner G.J. (2014). L1 retrotransposons and somatic mosaicism in the brain. Annu. Rev. Genet..

[115] Rippe K., Luke B. (2015). TERRA and the state of the telomere. Nat. Struct. Mol. Biol..

[116] Redon S., Reichenbach P., Lingner J. (2010). The non-coding RNA TERRA is a natural ligand and direct inhibitor of human telomerase. Nucleic Acids Res..

[117] Azhibek D., Skvortsov D., Andreeva A., Zatsepin T., Arutyunyan A., Zvereva M., Dontsova O. (2016). TERRA mimicking ssRNAs prevail over the DNA substrate for telomerase in vitro due to interactions with the alternative binding site. J. Mol. Recognit..

[118] Redon S., Zemp I., Lingner J. (2013). A three-state model for the regulation of telomerase by TERRA and hnRNPA1. Nucleic Acids Res..

[119] Kreilmeier T., Mejri D., Hauck M., Kleiter M., Holzmann K. (2016). Telomere transcripts target telomerase in human cancer cells. Genes.

[120] Wang Z., Lieberman P.M. (2016). The crosstalk of telomere dysfunction and inflammation through cell-free TERRA containing exosomes. RNA Biol..

[121] Arnoult N., Van Beneden A., Decottignies A. (2012). Telomere length regulates TERRA levels through increased trimethylation of telomeric h3k9 and hp1alpha. Nat. Struct. Mol. Biol..

[122] Porro A., Feuerhahn S., Reichenbach P., Lingner J. (2010). Molecular dissection of telomeric repeat-containing RNA biogenesis unveils the presence of distinct and multiple regulatory pathways. Mol. Cell. Biol..

[123] Deng Z., Wang Z., Stong N., Plasschaert R., Moczan A., Chen H.S., Hu S., Wikramasinghe P., Davuluri R.V., Bartolomei M.S. (2012). A role for CTCF and cohesin in subtelomere chromatin organization, TERRA transcription, and telomere end protection. EMBO J..

[124] Diman A., Boros J., Poulain F., Rodriguez J., Purnelle M., Episkopou H., Bertrand L., Francaux M., Deldicque L., Decottignies A. (2016). Nuclear respiratory factor 1 and endurance exercise promote human telomere transcription. Sci. Adv..

[125] Eid R., Demattei M.V., Episkopou H., Auge-Gouillou C., Decottignies A., Grandin N., Charbonneau M. (2015). Genetic inactivation of atrx leads to a decrease in the amount of telomeric cohesin and level of telomere transcription in human glioma cells. Mol. Cell. Biol..

[126] Episkopou H., Draskovic I., Van Beneden A., Tilman G., Mattiussi M., Gobin M., Arnoult N., Londono-Vallejo A., Decottignies A. (2014). Alternative lengthening of telomeres is characterized by reduced compaction of telomeric chromatin. Nucleic Acids Res..

[127] Benetti R., Garcia-Cao M., Blasco M.A. (2007). Telomere length regulates the epigenetic status of mammalian telomeres and subtelomeres. Nat. Genet..

[128] Benetti R., Gonzalo S., Jaco I., Munoz P., Gonzalez S., Schoeftner S., Murchison E., Andl T., Chen T., Klatt P. (2008). A mammalian microRNA cluster controls DNA methylation and telomere recombination via Rbl2-dependent regulation of DNA methyltransferases. Nat. Struct. Mol. Biol..

[129] Lu F., Liu Y., Jiang L., Yamaguchi S., Zhang Y. (2014). Role of tet proteins in enhancer activity and telomere elongation. Genes Dev..

[130] Tilman G., Loriot A., Van Beneden A., Arnoult N., Londono-Vallejo J.A., De Smet C., Decottignies A. (2009). Subtelomeric DNA hypomethylation is not required for telomeric sister chromatid exchanges in ALT cells. Oncogene.

[131] Yehezkel S., Segev Y., Viegas-Pequignot E., Skorecki K., Selig S. (2008). Hypomethylation of subtelomeric regions in icf syndrome is associated with abnormally short telomeres and enhanced transcription from telomeric regions. Hum. Mol. Genet..

[132] Montero J.J., Lopez de Silanes I., Grana O., Blasco M.A. (2016). Telomeric RNAs are essential to maintain telomeres. Nat. Commun..

[133] Vajen B., Modlich U., Schienke A., Wolf S., Skawran B., Hofmann W., Busche G., Kreipe H., Baum C., Santos-Barriopedro I. (2013). Histone methyltransferase Suv39h1 deficiency prevents myc-induced chromosomal instability in murine myeloid leukemias. Genes Chromosom. Cancer.

[134] Clynes D., Jelinska C., Xella B., Ayyub H., Scott C., Mitson M., Taylor S., Higgs D.R., Gibbons R.J. (2015). Suppression of the alternative lengthening of telomere pathway by the chromatin remodelling factor atrx. Nat. Commun..

[135] Schwartzentruber J., Korshunov A., Liu X.Y., Jones D.T., Pfaff E., Jacob K., Sturm D., Fontebasso A.M., Quang D.A., Tonjes M. (2012). Driver mutations in histone H3.3 and chromatin remodelling genes in paediatric glioblastoma. Nature.

[136] Chang F.T., Chan F.L., JD R.M., Udugama M., Mayne L., Collas P., Mann J.R., Wong L.H. (2015). CHK1-driven histone H3.3 serine 31 phosphorylation is important for chromatin maintenance and cell survival in human alt cancer cells. Nucleic Acids Res..

[137] Conomos D., Reddel R.R., Pickett H.A. (2014). Nurd-znf827 recruitment to telomeres creates a molecular scaffold for homologous recombination. Nat. Struct. Mol. Biol..

[138] Osterwald S., Deeg K.I., Chung I., Parisotto D., Worz S., Rohr K., Erfle H., Rippe K. (2015). PML induces compaction, TRF2 depletion and DNA damage signaling at telomeres and promotes their alternative lengthening. J. Cell Sci..

[139] Jamiruddin M.R., Kaitsuka T., Hakim F., Fujimura A., Wei F.Y., Saitoh H., Tomizawa K. (2016). HDAC9 regulates the alternative lengthening of telomere (ALT) pathway via the formation of alt-associated pml bodies. Biochem. Biophys. Res. Commun..

[140] Novo C.L., Polese C., Matheus N., Decottignies A., Londono-Vallejo A., Castronovo V., Mottet D. (2013). A new role for histone deacetylase 5 in the maintenance of long telomeres. FASEB J..

[141] Ng L.J., Cropley J.E., Pickett H.A., Reddel R.R., Suter C.M. (2009). Telomerase activity is associated with an increase in DNA methylation at the proximal subtelomere and a reduction in telomeric transcription. Nucleic Acids Res..

[142] Noelyn A.H., Janice A.R., Tania L.S. (2013). Molecular markers of glioblastoma and the potential for integration with imaging: The future for assigning prognosis and best treatment strategy. Curr. Mol. Imaging.

[143] Shirode A.B., Kovvuru P., Chittur S.V., Henning S.M., Heber D., Reliene R. (2014). Antiproliferative effects of pomegranate extract in MCF-7 breast cancer cells are associated with reduced DNA repair gene expression and induction of double strand breaks. Mol. Carcinog..

[144] Yang L., Li Y., Cheng M., Huang D., Zheng J., Liu B., Ling X., Li Q., Zhang X., Ji W. (2012). A functional polymorphism at microrna-629-binding site in the 3′-untranslated region of NBS1 gene confers an increased risk of lung cancer in southern and eastern chinese population. Carcinogenesis.

[145] Nergadze S.G., Farnung B.O., Wischnewski H., Khoriauli L., Vitelli V., Chawla R., Giulotto E., Azzalin C.M. (2009). Cpg-island promoters drive transcription of human telomeres. RNA.

[146] Arora R., Azzalin C.M. (2015). Telomere elongation chooses TERRA alternatives. RNA Biol..

[147] Tagami H., Ray-Gallet D., Almouzni G., Nakatani Y. (2004). Histone H3.1 and H3.3 complexes mediate nucleosome assembly pathways dependent or independent of DNA synthesis. Cell.

[148] Hoare S.F., Bryce L.A., Wisman G.B., Burns S., Going J.J., van der Zee A.G., Keith W.N. (2001). Lack of telomerase RNA gene hTERc expression in alternative lengthening of telomeres cells is associated with methylation of the hTERc promoter. Cancer Res..

[149] Atkinson S.P., Hoare S.F., Glasspool R.M., Keith W.N. (2005). Lack of telomerase gene expression in alternative lengthening of telomere cells is associated with chromatin remodeling of the hTR and hTERT gene promoters. Cancer Res..

[150] Christodoulidou A., Raftopoulou C., Chiourea M., Papaioannou G.K., Hoshiyama H., Wright W.E., Shay J.W., Gagos S. (2013). The roles of telomerase in the generation of polyploidy during neoplastic cell growth. Neoplasia.

[151] Conomos D., Stutz M.D., Hills M., Neumann A.A., Bryan T.M., Reddel R.R., Pickett H.A. (2012). Variant repeats are interspersed throughout the telomeres and recruit nuclear receptors in ALT cells. J. Cell Biol..

[152] Marzec P., Armenise C., Perot G., Roumelioti F.M., Basyuk E., Gagos S., Chibon F., Dejardin J. (2015). Nuclear-receptor-mediated telomere insertion leads to genome instability in ALT cancers. Cell.

[153] Rousseau P., Autexier C. (2015). Telomere biology: Rationale for diagnostics and therapeutics in cancer. RNA Biol..

[154] Shay J.W. (2016). Role of telomeres and telomerase in aging and cancer. Cancer Discov..

[155] Conomos D., Pickett H.A., Reddel R.R. (2013). Alternative lengthening of telomeres: Remodeling the telomere architecture. Front. Oncol..

[156] Liang J., Yang F., Zhao L., Bi C., Cai B. (2016). Physiological and pathological implications of 5-hydroxymethylcytosine in diseases. Oncotarget.

[157] Kraus T.F., Globisch D., Wagner M., Eigenbrod S., Widmann D., Munzel M., Muller M., Pfaffeneder T., Hackner B., Feiden W. (2012). Low values of 5-hydroxymethylcytosine (5hmC), the “sixth base”, are associated with anaplasia in human brain tumors. Int. J. Cancer.

[158] Lee D.H., Ryu H.W., Won H.R., Kwon S.H. (2017). Advances in epigenetic glioblastoma therapy. Oncotarget.

[159] Lee P., Murphy B., Miller R., Menon V., Banik N.L., Giglio P., Lindhorst S.M., Varma A.K., Vandergrift W.A., Patel S.J. (2015). Mechanisms and clinical significance of histone deacetylase inhibitors: Epigenetic glioblastoma therapy. Anticancer Res..

[160] Yang R., Wu Y., Wang M., Sun Z., Zou J., Zhang Y., Cui H. (2015). HDAC9 promotes glioblastoma growth via taz-mediated egfr pathway activation. Oncotarget.

[161] Yang Y.L., Huang P.H., Chiu H.C., Kulp S.K., Chen C.S., Kuo C.J., Chen H.D., Chen C.S. (2013). Histone deacetylase inhibitor AR42 regulates telomerase activity in human glioma cells via an Akt-dependent mechanism. Biochem. Biophys. Res. Commun..

[162] Hashizume R., Andor N., Ihara Y., Lerner R., Gan H., Chen X., Fang D., Huang X., Tom M.W., Ngo V. (2014). Pharmacologic inhibition of histone demethylation as a therapy for pediatric brainstem glioma. Nat. Med..

[163] Grasso C.S., Tang Y., Truffaux N., Berlow N.E., Liu L., Debily M.A., Quist M.J., Davis L.E., Huang E.C., Woo P.J. (2015). Functionally defined therapeutic targets in diffuse intrinsic pontine glioma. Nat. Med..

[164] Lee J., Solomon D.A., Tihan T. (2017). The role of histone modifications and telomere alterations in the pathogenesis of diffuse gliomas in adults and children. J. Neuro-Oncol..

[165] Vanan M.I., Underhill D.A., Eisenstat D.D. (2017). Targeting epigenetic pathways in the treatment of pediatric diffuse (high grade) gliomas. Neurotherapeutics.

[166] Williams M.J., Singleton W.G., Lowis S.P., Malik K., Kurian K.M. (2017). Therapeutic targeting of histone modifications in adult and pediatric high-grade glioma. Front. Oncol..

[167] Ferreira W.A., Pinheiro Ddo R., Costa Junior C.A., Rodrigues-Antunes S., Araujo M.D., Leao Barros M.B., Teixeira A.C., Faro T.A., Burbano R.R., Oliveira E.H. (2016). An update on the epigenetics of glioblastomas. Epigenomics.

[168] Adams B.D., Parsons C., Walker L., Zhang W.C., Slack F.J. (2017). Targeting noncoding RNAs in disease. J. Clin. Investig..

[169] Rupaimoole R., Slack F.J. (2017). Microrna therapeutics: Towards a new era for the management of cancer and other diseases. Nat. Rev. Drug Dis..

[170] Swierczynski S., Klieser E., Illig R., Alinger-Scharinger B., Kiesslich T., Neureiter D. (2015). Histone deacetylation meets miRNA: Epigenetics and post-transcriptional regulation in cancer and chronic diseases. Expert Opin. Biol. Ther..

[171] Kasinski A.L., Slack F.J. (2011). Epigenetics and genetics. Micrornas en route to the clinic: Progress in validating and targeting microRNAs for cancer therapy. Nat. Rev. Cancer.

[172] Kim M., Kasinski A.L., Slack F.J. (2011). Microrna therapeutics in preclinical cancer models. Lancet Oncol..

[173] Preusser M., Marosi C. (2017). Neuro-oncology in 2016: Advances in brain tumour classification and therapy. Nat. Rev. Neurol..

